# Analysis of Wheat Prolamins, the Causative Agents of Celiac Sprue, Using Reversed Phase High Performance Liquid Chromatography (RP-HPLC) and Matrix-Assisted Laser Desorption Ionization Time of Flight Mass Spectrometry (MALDI-TOF-MS)

**DOI:** 10.3390/nu6041578

**Published:** 2014-04-15

**Authors:** Jaime H. Mejías, Xiaoqiao Lu, Claudia Osorio, Jeffrey L. Ullman, Diter von Wettstein, Sachin Rustgi

**Affiliations:** 1 Department of Crop & Soil Sciences, Washington State University, Pullman, WA 99164, USA; E-Mails: mejias.j@gmail.com (J.H.M.); xiaoqiao.lu@wsu.edu (X.L.); cosorio72@gmail.com (C.O.); 2 Department of Agricultural and Biological Engineering, University of Florida, Gainesville, FL 32611, USA; E-Mail: jullman@ufl.edu; 3 School of Molecular Biosciences, Washington State University, Pullman, WA 99164, USA; 4 Center for Reproductive Biology, Washington State University, Pullman, WA 99164, USA

**Keywords:** prolamins, glutenin, gliadin, celiac disease, wheat

## Abstract

Wheat prolamins, commonly known as “gluten”, are a complex mixture of 71–78 proteins, which constitute ~80% of the proteins in the wheat grains and supply 50% of the global dietary protein demand. Prolamins are also responsible for numerous gluten-induced disorders and determine the unique visco-elastic properties of the wheat dough. These properties necessitate the reliable determination of the prolamin composition in wheat grains and their derived products. Therefore, this study examined the impact of HPLC conditions, including column type, column temperature, flow rate, and the gradient of polar and non-polar solvents in the mobile phase, to improve the analytical resolution of prolamins. The following conditions were found optimal for analyses: column temperature 60 °C, flow rate 1.0 mL/min and an elution gradient of 20%–60% of 0.1% trifluoroacetic acid + acetonitrile in 60 min. For further improvement of resolution, gliadin and glutenin extracts were analyzed using MALDI-TOF-MS in combination with HPLC fractionation. Two semi-quantitative methods, densitometry of stained polyacrylamide gels and HPLC, were used to determine relative prolamin quantities and the correspondence between the methods was established. The combinatorial gluten analyses approach developed during the present study was used to analyze prolamin profiles of wheat transformants expressing *DEMETER* silencing artificial microRNA, and the results are discussed.

## 1. Introduction

Wheat supplies about 20% of the total food calories consumed worldwide and is a national staple in many countries [1]. In the U.S., the per capita consumption of wheat exceeds that of any other single food staple [2]. Despite serving as a major energy source, wheat is also a major cause of frequent diet-induced health issues, including celiac disease, gluten sensitivity, and food allergies [3,4,5,6]. Among these disorders, celiac disease is the most common food-born enteropathy in humans, occurring in the U.S. with an incidence of 1 in 100 [6]. Due to improvements in disease diagnostics and adaptation to a gluten-rich dietary pattern, it is increasingly being diagnosed in apparently “celiac-free” areas of the world [7].

Celiac disease is an inheritable life-long enteropathy of the proximal small intestine. The disease is triggered by consumption of gluten, a group of storage proteins found in Triticeae grains, which include wheat, barley, and rye. These proteins promote intestinal inflammation, villous atrophy, and crypt cell hyperplasia, which lead to a significant decrease in intestinal wall surface area. As a consequence, a number of symptoms affect celiac patients, such as diarrhea, weight loss, anemia, and bone disorders [8,9].

Gluten is a complex mixture of proteins with unique biochemical properties that allow the formation of the visco-elastic mass of polypeptides essential for dough and pasta formation [10,11]. These proteins are also used broadly in the food industry as additives to sauces, soups, and canned meals [9]. Gluten proteins consist of a mixture of monomeric gliadins and polymeric glutenin subunits, which are present in approximately equal amounts. Together they form 80% of the total storage protein content in the wheat kernel [12]. The remainder is albumins (12%) and globulins (8%) [13]. The highly homologous seed storage proteins of wheat and barley are classified on the basis of their solubility into water-soluble albumins (soluble in diluted neutral buffers), salt-soluble globulins, alcohol-soluble (40%–70%) gliadins, and alcohol-insoluble glutenins (solubilize in alcohol only under reducing conditions) [14]. In wheat, the alcohol insoluble fraction of prolamins is comprised of the High Molecular Weight (HMW) glutenin subunits (650–850 residues long) and the Low Molecular Weight (LMW) glutenin subunits (270–330 residues long), whereas the alcohol soluble fraction is comprised of gliadins (250–720 residues long) [9,14]. The latter can be separated into sulphur-poor ω-gliadins and the sulphur-rich α/β and γ gliadins. In barley the homologous proteins are named D-, C-, B- and γ-hordeins, respectively [9]. Glutamine (Gln or Q) and proline (Pro or P) are two major amino acids that comprise 35 and 15% of the gluten proteins, respectively; hence, these proteins are also dubbed as prolamins [15]. The amino acid composition of these polypeptides makes them highly resistant to gastric and pancreatic proteases. The partially digested polypeptides move from the stomach into the intestine, where they are incompletely hydrolyzed by the exopeptidases of the brush border membrane [16,17,18,19,20]. The antigenicity of these peptides is intensified by deamidation of selected glutamine residues by tissue transglutaminase 2 (tTG2), which increases their affinity to the human leukocyte antigen (HLA)-DQ2 or HLA-DQ8 [16]. The peptide-DQ2 complex enhances the inflammatory gut mucosa response, resulting in an increase of Th1 cells [20,21,22,23], which ultimately leads to painful chronic erasure of intestinal microvilli in patients with active celiac disease.

In view of the importance of gluten proteins as major determinants of end-use quality, and also the cause of frequent diet-induced health issues. Procedures for their quantification and biochemical characterization have been developed in the past. However, most of the procedures described in the literature exhibit low reproducibility, poor resolution and/or are labor intensive and time consuming. This instigates a need for standardization and improvement of extraction and separation procedures based on existing techniques of electrophoresis, chromatography and mass spectrometry, as well as for rapid and reproducible characterization of individual prolamins.

The objective of this study was to evaluate the influence of analytical conditions using HPLC and MALDI-TOF-MS procedures for analysis of wheat prolamins. A comparison between HPLC and densitometric method was also performed to evaluate differences in the gluten quantification efficacies of the two procedures. Better documentation of the impacts of various method conditions can be used to optimize the detection and characterization of prolamins. The results obtained by this study complement various reported methods (*cf*. [24,25,26,27,28,29,30]), and provide a valuable reference to researchers performing analysis of glutens. Refined analytical methods will not only be beneficial for protein profiling of different wheat/barley varieties, but also will be useful for testing the level of contamination in allegedly “gluten-free” products and characterizing mutants/transformants lacking prolamins.

## 2. Materials and Methods

### 2.1. Reagents and Chemicals

All solvents and chemicals used for sample preparations were either HPLC grade or analytical quality, unless stated otherwise. The dithiothreitol and bovine serum albumin were purchased from Sigma-Aldrich, St. Louis, MO, USA. For the preparation of HPLC solvents, trifluoroacetic acid and acetonitrile were purchased from MP Biomedicals, Santa Ana, CA, USA.

### 2.2. Plant Material

Seeds of the soft white winter wheat cultivar Brundage 96 were procured from the variety testing program located at the Washington State University (Pullman, WA, USA) and seeds of the spring wheat cultivar Bobwhite “S” (PI 520368) were procured from the National Small Grains Collection (Aberdeen, ID, USA). Brundage 96 is well adapted to the U.S. Pacific Northwest, has excellent cookie and sponge cake making quality, and is used as explant donor in the transformation experiments performed by us to develop wheat lines showing RNA interference against *DEMETER* homoeologues [12]. Whereas Bobwhite is a model cultivar used frequently in laboratory experiments, it is also well known for high transformation efficiency.

### 2.3. Protein Extraction

Mature kernels of Brundage 96 and Bobwhite were ground to fine powder (flour) with a pestle and mortar. A sequential extraction procedure to separate albumins/globulins, gliadins and glutenins was adopted (*cf*. [24]). Briefly, the albumins/globulins were extracted twice from 200 mg of non-defatted flour with 1 mL of 0.4 M NaCl by incubation in an orbital shaker at ambient temperature with gentle shaking (200 rpm) for 20 min followed by centrifugation at 10,000× *g* for 20 min at room temperature. The supernatant containing the albumin/globulin fraction was collected and saved. The gliadin fraction from the remaining pellet was extracted stepwise three times with 500 μL of 60% (v/v) ethanol, incubated with gentle shaking (200 rpm) for 20 min at room temperature, and centrifuged for 20 min at 10,000× *g*. Following centrifugation the supernatant containing the gliadin fraction was collected. Subsequently the glutenin fraction was obtained by extracting the pellet from the previous step two times with 1 mL of 50% (v/v) aqueous 1-propanol, 50 mM Tris-HCl (pH 7.5) and 2% (w/v) dithiothreitol for 20 min by incubation at 53 °C with gentle shaking (200 rpm). The suspension was then centrifuged at 12,000 and 14,000× *g* for the first and second rounds of extraction, respectively. The gliadin and glutenin extracts were passed through 0.45 μm pore size Durapore^®^ 13 mm membrane filters (EMD Millipore Corporation, Billerica, MA, USA).

The gliadin and glutenin fractions were quantified using Quick Start Bradford 1× dye reagent (Cat # 500–0205, Bio-Rad Laboratories, Hercules, CA, USA) following the manufacturer’s instructions. Six known dilutions of bovine serum albumin from 0.2 to 2.2 μg/mL were used as standards, and absorbance was measured at 595 nm on a BioRad SmartSpec Plus spectrophotometer (Bio-Rad Laboratories, Hercules, CA, USA). Linear regression was performed using the resulting values to calculate the amount of gliadins and glutenins in each sample. The principle of the assay is based on the binding of Coomassie Brilliant Blue G-250 dye (Sigma-Aldrich, St. Louis, MO, USA) to proteins [31]. The dye exists in three forms: cationic, neutral and anionic with absorbance, respectively in the red, green and blue part of the visible spectrum. When the dye binds to protein, it gets converted to a stable un-protonated blue form with a maximum absorbance at 595 nm.

### 2.4. Polyacrylamide Gel Electrophoresis (PAGE)

Gliadins represent a heterogeneous mixture of 45–50 single-chain polypeptides with overlapping masses in common wheat pure lines, which require acidic-native polyacrylamide gels to achieve the desired resolution. The native-polyacrylamide gels maintain the conformation of the loaded protein(s) unaltered, allowing for their separation based on physicochemical properties. Glutenins, on the other hand, exist in the form of cross-linked proteins and in common wheat pure lines contain up to 23 LMW glutenin subunits and 3–5 HMW glutenin subunits, which upon reduction can be resolved on denaturing SDS-polyacrylamide gels. Thus, in the present communication gliadins were resolved on lactic acid PAGE and glutenins on SDS-PAGE. The protocols used for lactic acid and SDS-polyacrylamide gels are as follows.

#### 2.4.1. Acid-PAGE

The method used for acid-PAGE was modified from Khan *et al.* [32]. Briefly, the gel solution containing 5.7% of acrylamide and 0.28% of *NN*-ethylene-bis-acrylamide was prepared by diluting commercially available stock solutions of 40% acrylamide (Cat # 161-0140, Bio-Rad Laboratories, Hercules, CA, USA) and 2% bis-acrylamide (Cat # 161-0142, Bio-Rad Laboratories, Hercules, CA, USA) in 1× aluminium lactate buffer (containing 4.25 μM aluminum l-lactate (Cat # 25301, Sigma-Aldrich, St. Louis, MO, USA) (pH 3.1). The l-ascorbic acid (Cat # A5960, Sigma-Aldrich, St. Louis, MO, USA) and Iron (II) sulfate 7-hydrate (Cat # 131362, Panreac Química S.L.U., Barcelona, Spain) were added to 30 mL of the solution containing acrylamide and bis-acrylamide at the final concentrations of 0.238 mg/mL and 2 × 10^−3^ mg/mL, respectively. Just before casting, the solution was supplemented with 30% hydrogen peroxide (Cat # 2186-01, Avantor Performance Materials, Center Valley, PA, USA) at a final concentration of 0.03%.

Samples were prepared by adding 5 μL of 5× loading buffer (containing 5 g sucrose and 2 mg of methyl violet in 10 mL of water) to 5 μL of gliadin solution before loading. The samples were loaded on the gel and electrophoresed at 500 volts (40 mA/gel) for 120 min using Amersham Biosciences’s SE Ruby 600 system (GE Healthcare Bio-Sciences, Pittsburgh, PA, USA). However, when gliadin traces (*i.e*., fractions collected at different time intervals from HPLC in 50 mL falcon tubes) were loaded onto the acid-PAGE, the fractions were first lyophilized for two days and later re-suspended in 100 μL of 60% ethanol before loading. After the electrophoresis run, the gels were equilibrated for 30 min in 10% trichloroacetic acid (TCA). Following equilibration, 5 mL of Coomassie Brilliant Blue R-250 solution [prepared by dissolving 25 mg of Brilliant Blue R-250 (Cat # BP101-25, Thermo Fisher Scientific Inc., Waltham, MA, USA) in 5 mL of 96% ethanol] was applied to each gel. Gels were stained with gentle shaking for 24 h at room temperature and de-stained in distilled water (containing 10 drops of Triton 100) for 30 min. After staining, the gels were visualized and pictured using Kodak image system (Molecular Bioimaging, Alta Loma, CA, USA).

#### 2.4.2. Sodium Dodecyl Sulfate-PAGE (SDS-PAGE)

To resolve reduced glutenin subunits, 12% SDS-PAGE was prepared following Fling and Gregerson [33]. Briefly, the gel is comprised of two layers: the separating layer and the stacking layer. The separating gel was prepared by mixed 4.2 mL of acrylamide stock solution (30% acrylamide: 0.8% bis acrylamide; Cat #161-0154, Bio-Rad Laboratories, Hercules, CA, USA) with 4.2 mL of water, 3 mL of 3M Tris-HCl (pH 8.8), 120 μL of 10% SDS, 120 μL of 10% ammonium persulfate (APS) and 6 μL of tetramethylethylenediamin (TEMED). After polymerization, the separating gel was layered with the stacking gel prepared using 1 mL of acrylamide solution, 750 μL 1M Tris-HCl (pH 6.8), 4.25 mL of water, 60 μL of 10% SDS, 60 μL 10% APS, and 4 μL of TEMED.

The gels were electrophoresed on a BioRad Mini-PROTEAN^®^ 2-gel electrophoresis system (Cat# 165-8003, Bio-Rad Laboratories, Hercules, CA, USA) using a electrophoresis buffer containing 25mM Tris-HCL, 192mM glycine and 0.1% SDS at 120 volts for 2 h. Following electrophoresis, the gels were stained in colloidal Coomassie stain following Neuhoff *et al.* [34]. The glutenin samples were prepared by acetone precipitation followed by resuspension in 200 μL of 1-propanol, and 5 μL of this concentrate was used for loading on the gels. A pre-stained protein molecular weight marker (Cat # SM0441, Thermo Fisher Scientific Inc., Rockford, IL, USA) was loaded on each gel for size estimation.

### 2.5. Reversed-Phase High-Performance Liquid Chromatography (RP-HPLC)

The HPLC separations were performed using a C8 reversed-phase analytical column (Zorbax 300SB-C8, Agilent Technologies, Palo Alto, CA, USA) with 5 μm particle size and 30 nm microporous silica diameter (250 mm length, 4.6 mm inner diameter) on a 1200 Series Quaternary HPLC-System (Agilent Technologies, Palo Alto, CA, USA), with a diode array UV-Vis detector. Column temperature was kept at 60 °C. A linear elution gradient was implemented using two mobile solvents: the polar solvent A consisting of 0.1% trifluoroacetic acid (v/v) in type I ultrapure water (18 MΩ·cm specific resistance) and the non-polar solvent B containing 0.1% trifluoroacetic acid (v/v) and acetonitrile. Absorbance was monitored at a detection wavelength of 210 nm, and the flow rate was maintained at 1.0 mL min^−1^. The elution gradient conditions were selected as follows: for gliadins a linear gradient from 20% to 60% B for 60 min, and for glutenins from 0% to 24% B for 20 min followed by 24%–60% B for 40 min. After each run, the column was cleared with 90% B for 3 min, and equilibrated with the starting B concentration for 5 min.

Gliadin fractionation was attained by injecting 90 μL of each sample into the system, and three fractions at retention times of 13–25 min, 25–33 min, and 33–44 min were collected manually in 50 mL centrifuge tubes. Glutenin fractionation was attained by injecting 80 μL of sample, and six fractions at retention times of 26–29 min, 31.5–36 min, 38–43 min, 43–46 min, 46–48.5 min, and 48.5–53 min were collected.

The fractions were collected by connecting a Teflon^®^ tube (220 mm length and 0.5 mm inner diameter, Cole-Parmer, Vernon Hills, IL USA) to the waste outlet located after the Diode Array UV-Vis detector. A 2.6 sec delay between detection and the collection point was calculated based on the flow rate, the tubing length and internal cross-sectional area. Each run was repeated three times. RP-HPLC chromatograms showed no variation between runs (Figure S1), consequently the fraction volume of each run was combined to increase the amount of protein in the final sample (Figure S1). Samples were immediately frozen at −80 °C for 24 h and lyophilized at −60 °C for 48 h. Freeze-dried samples were stored at room temperature (21 °C) before SDS- and acid-PAGE analyses.

### 2.6. Matrix-Assisted Laser Desorption/Ionization Time-of-Flight Mass Spectrometry (MALDI-TOF-MS)

MALDI-TOF-MS was used to obtain the mass spectra of the bulked gliadin and glutenin extracts from Brundage 96, and also of their HPLC traces (fractions) collected at different retention times. The bulk samples were used as such, whereas the HPLC fractions collected at different retention times were freeze-dried and re-suspended in 100 μL of 60% ethanol. The freeze-drying was preferred over the vacuum drying to concentrate the HPLC fractions. As the freeze-drying is based on the solid to gas transition (*i.e.*, sublimation), which is gentler than the liquid to gas transition (*i.e.*, evaporation) that take place during vacuum drying in a cold-trap or speed-vac, it is far less likely to cause oxidative damage to the protein samples. In addition, the vacuum drying is more suitable for the samples with small volumes than for larger volumes of material. 

All samples, including the HPLC fractions and the bulked gliadins and glutenins, were mixed in 1:1 proportion with sinapic acid dissolved in 50% acetonitrile containing 0.1% trifluoroacetic acid. About 0.3 μL of this mixture was layered over the spot prepared using the sinapic acid matrix. The mass spectra of each sample was recorded on a AB SCIEX 4800 MALDI TOF/TOF Analyzer (AB SCIEX, Framingham, MA, USA) using the linear high mass positive method at an acceleration voltage of 20 kV and 1400 ns delay time by capturing 500 spectra of a single laser shot. Each sample was replicated twice using the MALDI TOF/TOF to avoid experimental errors. The equipment was calibrated using singly and doubly charged signals of bovine serum albumin (BSA) with a molecular mass of 66.43 and 33.215 kDa, respectively.

### 2.7. Relative-Quantification of Gliadin and Glutenin Fractions

Two procedures, densitometry of stained polyacrylamide gels and HPLC, were applied to determine relative quantities of gliadins and glutenins in a sample. The correspondence between the values obtained using the two methods was determined.

#### 2.7.1. Standard Curve Development Using Densitometry

To translate optical density into protein concentration in μg/mL, a standard curve was prepared using optical densities determined from a scanned image of a Coomassie stained SDS-polyacrylamide gel loaded with known quantities (0, 2.5, 5, 10, 25, 50, 75, 100, 150, and 300 μg/mL) of BSA. The gel was scanned using a Kodak Image Station 4000 MM (Molecular Bioimaging, Alta Loma, CA, USA) and analyzed using the “Gel Analyzer” module of the ImageJ software version 1.45 s [35]. The optical densities were determined for each protein band after transforming the scanned image to 8-bits and subtracting the image background. The optical density (OD) values plotted as peaks correspond with the intensities of the protein band in each lane of the polyacrylamide gel. The peak area was recorded and correlated with the BSA concentration loaded onto the gel. A standard curve was obtained using a linear regression model. Analysis of an acid-polyacrylamide gel for gliadins and a SDS-polyacrylamide gel for glutenins was conducted in a similar fashion as for BSA, and the peak area for each band was calculated. These values were plotted on the standard curve to determine relative protein concentrations, and the values obtained for each band were summed to calculate a cumulative concentration for the respective glutenin and gliadin fractions.

#### 2.7.2. Standard Curve Development Using HPLC

Development of a standard curve for the HPLC procedure was conducted through the use of increasing BSA concentrations (0, 10, 25, 50, 75, 100, 150, and 300 μg/mL) that were loaded onto the C8 reversed-phase analytical column (Zorbax 300SB-C8, Agilent Technologies, Palo Alto, CA, USA). A linear elution gradient was implemented using two mobile solvents, the polar solvent A consisting of 0.2% trifluoroacetic acid (v/v) in type I ultrapure water (18 MΩ·cm specific resistance), and the non-polar solvent B containing 0.15% trifluoroacetic acid (v/v) and acetonitrile. Absorbance was monitored at a detection wavelength of 280 nm, column temperature was kept at 40 °C, and the flow rate was maintained at 1.0 mL min^−1^. A linear gradient from 26% to 55% B in 30 min was implemented following Long [36]. Two peaks were observed respectively at 16.78 and 17.98 min retention times. Peaks were integrated and the obtained peak area in milli-absorbance unit (mAU) was regressed against the concentrations of BSA loaded onto the HPLC column to obtain a standard curve. The gliadins and glutenins were analyzed in analogous fashion as mentioned above for BSA and the peak areas were calculated. The mAU values were plotted on the standard curve to determine relative protein concentrations, and the values obtained for each peak were summed to calculate a cumulative concentration for the respective glutenin and gliadin fractions.

## 3. Results and Discussion

Wheat prolamins form a heterogeneous mixture of seed storage proteins comprised of monomeric gliadins and polymeric glutenins. The other proteins stored in grains represent albumins and globulins [37]. These proteins include metabolically active enzymes, their precursors or structural proteins, and they are present in trace quantities except for α-/β-amylases and trypsin inhibitors in the albumin fraction and triticins in the globulin fraction [38]. Albumins and globulins are extracted using water and salt solutions, are less hydrophobic and are smaller in size than prolamins. These proteins cumulatively represent a very small fraction of the stored proteins, negligibly influence quality parameters, and are of minor nutritional interest; hence, these fractions are not discussed further. Based on their physical properties, like solubility, electrophoretic mobility, amino acid composition and relative mass (Mr) the gliadins and glutenins can be classified into alcohol soluble sulfur-poor α/β- and γ-gliadins and sulfur-rich ω-gliadins, and alcohol insoluble high and low molecular weight-glutenins [37]. The other factors that add to the complexity of the prolamins are products of gene duplication (these proteins are encoded by multi-gene families located on group 1 and 6 chromosomes), mutations and polyploidy, which result in allelic diversity, sequence homology and homoplasy [39]. This makes wheat/barley/rye prolamins the most complex proteins to resolve and characterize [40]. Thus, most of the traditional analytical methods are unsuitable for characterization of these proteins, necessitating development and optimization of alternative techniques [40]. Due to complexity of cereal proteins, a multistep extraction procedure is used and recommended [13].

Availability of HPLC columns that can resolve proteins provided new tools to the cereal chemist, and since their inception they have extensively been utilized to analyze plant proteins including prolamins (*cf*. [40]). Thus, in view of the benefits of using RP-HPLC for characterization of wheat prolamins, the present communication highlights the advantages of RP-HPLC combined with mass spectrometry to develop and optimize a fast and reliable platform for high-resolution analysis of wheat prolamins.

### 3.1. Parameter Optimization for RP-HPLC

The following parameters were tested to identify optimal analytical conditions to resolve wheat gliadins: two different wide pore “protein” columns (C8 and C18), three different column temperatures, four different flow rates, and two different gradients. The selection of the columns, which contain different packaging materials, was based on previous reports where both C18 and C8 columns were recommended to resolve wheat gliadin and glutenin fractions (*cf.* [40]). The Zorbax Eclipse Plus C18 packing is specifically designed to deliver high efficiency and excellent peak shape with all sample types [41]. This material was developed by first chemically bonding a dense monolayer of dimethyl-n-octadecylsilane stationary phase to a specially prepared ultra-high purity (>99% SiO_2_), ZORBAX Rx-SIL porous silica support that has a surface area of 160 m^2^/g and a controlled pore size of 95 Å. The column dimensions are 4.6 mm (inner diameter) × 150 mm (length) and the particle size is 5 μm. The Zorbax 300SB-C8 microparticulate column packing was specifically designed for RP-HPLC of peptides and proteins. The Stable Bond (SB) packing is made by chemically bonding a sterically protected octyl stationary phase to an ultra-high-purity, Zorbax, porous-silica microsphere that has a wide-pore size of 300 Å. The column dimensions are 4.6 mm (inner diameter) × 250 mm (length) with 30 nm microporous silica diameter and 5 μm particle size. The wide-pore size packing used in Zorbax 300SB-C8 columns is highly recommended for solutes with molecular sizes greater than 4 kDa; thus is highly suitable for analysis of prolamins with a relative mass range of 30–88 kDa. Two and four different flow rates were tested on C18 and C8 columns, respectively, by injecting 30 μL of Bobwhite gliadin extract using a linear gradient from 20 to 60% B for 60 min. In view of the column length differences, flow rates of 0.2 mL/min and 1.0 mL/min were applied on the C18 column and flow rates of 0.5 mL/min, 0.8 mL/min, 1.0 mL/min and 1.5 mL/min were applied on the C8 column. The C8 column always yielded better resolution at all flow rates compared to the C18 column, and the 1.0 mL/min flow rate exhibited the best results on the C8 column (Figure S2). A similar conclusion about the column choice was previously reached by Marchylo *et al.* [42], where sterically protected wide-pore monofunctional-silane bonded C8 and CN columns (Zorbax RX-300, Agilent Technology, Palo Alto, CA, USA) exhibited better gliadins and glutenins resolution compared to other silica-based columns.

As expected, negative correlations between the flow rate and the retention time, and the flow rate and the amount of eluent were observed. Though these properties can be favorably used in specific conditions, the lower flow rates require more solvents and operating time to elute proteins, which adds to the cost of each run and does not offer any obvious advantage in terms of resolution. Therefore, lower flow rates are not recommended for prolamin analysis.

Based on the manufacturer’s recommendations (40–65 °C) and previous reports, three column temperatures (40 °C, 50 °C and 60 °C) were tested using Bobwhite gliadin extracts on the C8 column (Figure S3). The best resolution was achieved at 60 °C at a flow rate of 1.0 mL/min with a linear gradient from 20% to 60% B for 60 min (Figure S3). This observation is in accordance with the previous findings, where Bietz and Cobb [43] observed marked improvement in gliadin resolution between 50 and 70 °C. Additionally, a positive correlation between an increase in column temperature and the extent of separation was generally observed [40]. This increase in resolution at higher column temperatures can be explained by the fact that a higher column temperature lowers the viscosity of the mobile phase, which results in increased diffusion of solutes from the stationary to the mobile phase [44]. The higher column temperatures can also be used to reduce the run time, because better chromatographic separation can be obtained at higher flow rates than those optimal at ambient temperatures [44]. Nevertheless, in view of the durability of the column, a temperature within the manufacturer’s recommended range is advisable.

As reported earlier, most of the proteins elute in the hydrophobicity range of 15%–80% aqueous acetonitrile (+0.1% trifluoroacetic acid) [40]. Thus, two different gradients, from 10% to 80% of solvent B and 20%–60% of solvent B, for 60 min were tested on a C8 column maintained at 60 °C using Bobwhite gliadin extract (Figure S4). The later gradient exhibited a clear advantage in terms of resolution over the former, and is recommended for analysis of wheat gliadins (Figure S4). The acetonitrile was used to make the aqueous gradient as it has low viscosity and is relatively transparent at 210 nm. Even though the ion-pairing agent trifluoroacetic acid can show relatively high absorbance at 210 nm, it is used in trace quantities (0.1%) and thus should not interfere with the analyses.

In summary, the following conditions were identified to best resolve wheat gliadins and were applied for all further analyses in this study: injection volume of 30 μL, column temperature of 60 °C, and a flow rate of 1.0 mL min^−1^ with an elution gradient of 20%–60% of solvent B for 60 min.

### 3.2. HPLC Fractionation and Mass Spectrometry for High-Resolution Analysis of Wheat Prolamins

Mass spectrometry (MS) gained popularity as a primary protein characterization method following the advent of soft desorption/ionization methods like electrospray ionization and matrix-assisted laser desorption/ionization (MALDI) in the late 1980s [45]. The superiority of these MS methods lies in their capacity to manage mixtures of intact proteins and peptides ranging in masses from 1000–100,000 Da without requiring chromatographic purification, low detection limits (in picomol range) and a detection speed of a few minutes [14,46]. Mass spectrometry is the only non-immunological method currently available for the detection of prolamins (gliadins and glutenins) in flours and food samples with a detection limit of 0.01 mg/mL, which is significantly lower than the detection limits of a sandwich ELISA used for this purpose [14,47]. The MALDI-TOF-MS can be divided into three components: binding of prolamins to the matrix, ionization and desorption of the prolamins by a laser separation (nitrogen gas UV laser with a wavelength of 337 nm, repetition rate of 1–20 Hz and pulse energy of 120–200 μJ), and detection of the prolamins by a mass spectrometer.

While RP-HPLC provides good resolution of wheat gliadin and glutenin fractions sufficient for varietal identification and/or characterization of genotypes, MALDI-TOF-MS has been increasingly used to improve analytical resolution. Although this technique was used to determine gluten contamination in food as early as 1995, it has only recently gained popularity [26,48,49]. Previous studies conducted to determine gluten contamination in food or performed to determine end-use product quality used bulked gliadin/glutenin extracts for analysis [14,46]. In this study, we performed MS analysis of gliadin and glutenin extracts of Brundage 96 in two different ways: (i) using bulk extracts; and (ii) using HPLC fractions. The bulked gliadin extract gave a high quality mass spectrum with 17 peaks ranging from 24.37 to 51.30 kDa, which represent various gliadin groups (Figure S5, Tables S1 and S2). However, when bulked glutenins were extracted using the traditional extraction buffer (50% 1-propanol, 2 M Urea, 0.05 M Tris-HCl (pH 7.5), 2% (w/v) dithiothreitol) an increase in background noise was observed during analysis (Figure S6). Thus, in view of the chaotropic properties of the urea, we decided to exclude urea from the extraction buffer. Glutenin extracts with and without urea were loaded on SDS-PAGE gel and their subsequent migration was observed to determine the effect of urea on glutenin extraction (Figure S7). Interestingly, no apparent effect of excluding urea from the extraction buffer was observed on the SDS-PAGE gel. The gluten extracted without urea was also analyzed using MALDI-TOF-MS. The gluten extract without urea showed improved signal-to-noise ratio, but none of the samples with/without urea yielded any protein detected beyond 42 kDa. Various proteins detected in the analysis ranged from 30 to 42 kDa, which falls within the known mass range for LMW glutenin subunits, and nothing was detected in the higher mass range of 65–90 kDa known for HMW glutenin subunits (Table S3).

**Figure 1:**
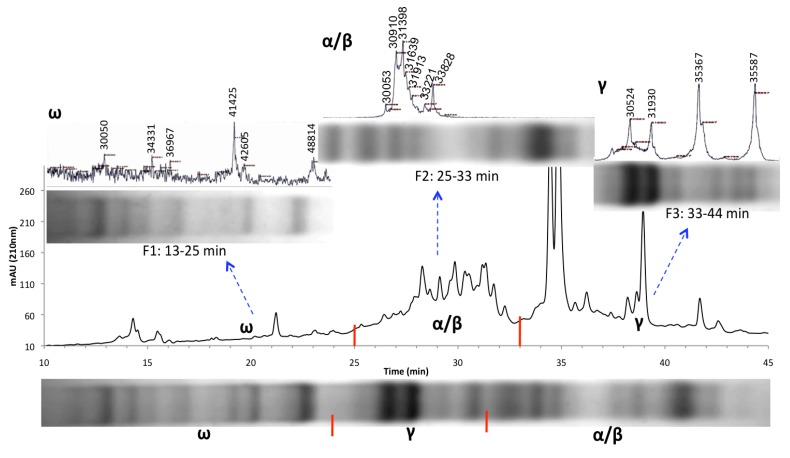
Mass spectra and electrophoresis patterns of the Brundage 96 gliadin extract, representing analysis of the bulk sample (**bottom**) and separate analysis of three HPLC fractions collected at 13–25, 25–33 and 33–44 min (**top**). Samples were resolved on acid-PAGE gels, as well as by MALDI-TOF-MS. F = fraction.

**Figure 2:**
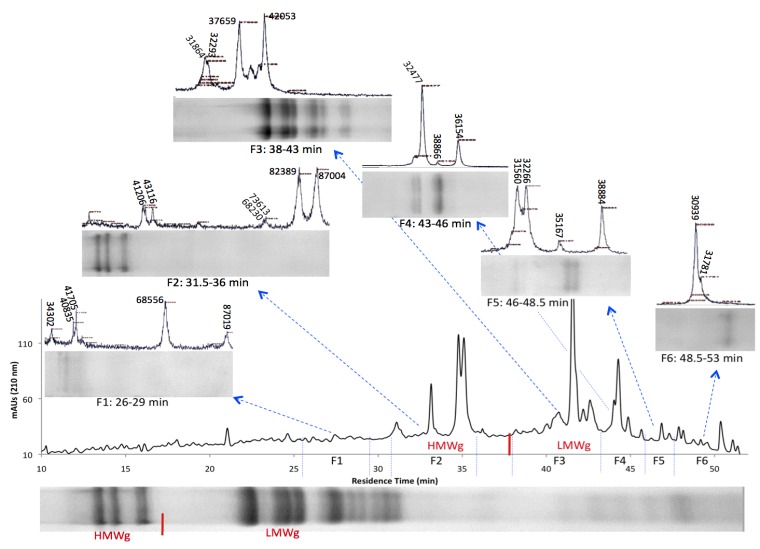
Mass spectra and electrophoresis patterns of the Brundage 96 glutenin extract, representing analysis of the bulk sample (**bottom**) and separate analysis of six HPLC fractions collected at 26–29, 31.5–36, 38–43, 43–46, 46.0–48.5 and 48.5–53.0 min (**top**). Samples were resolved on SDS-PAGE gels, as well as by MALDI-TOF-MS. F = fraction.

Bulk gliadin and glutenin extracts were respectively separated into three and six HPLC fractions to reduce spectral complexity, improve resolution in the higher mass range and remove matrix interference. Gliadin fractions were collected at the following three time intervals: 13–25, 25–33 and 33–44 min and glutenin fractions were collected at the following six time intervals 26–29, 31.5–36, 38–43, 43–46, 46–48.5 and 48.5–53 min (Figure 1, Figure 2 and Figure S1). Each HPLC fraction was freeze-dried to near completion and then re-suspended in 60% ethanol before MALDI-TOF-MS analysis. The number of peaks observed in the mass spectrum of each HPLC fraction and their molecular masses are summarized in Tables S4 and S5. Many more proteins were detected in the HPLC fractions compared to the bulked gliadin/glutenin extracts due to a reduction in matrix interference and spectral complexity. Thus, analysis of individual fractions significantly improved the resolution of protein analysis. For gliadins, 17 peaks were detected in the bulked sample and 44 peaks were detected when the three HPLC fractions were analyzed separately (Figure 1). Similarly, for glutenins 14 peaks were detected in the bulked sample and 40 peaks were detected when the six HPLC fractions were analyzed separately (Figure 2). In addition, glutenin fractions F1 and F2 yielded peaks belonging to HMW glutenin subunits, which escaped detection in the bulked sample. This demonstrates that the present analysis method has a significant advantage over traditional techniques where bulk gliadin and glutenin fractions were used for analyses. The use of this combinational approach for quantitative MS analysis is superior for determination of gluten contamination in food products, characterization of prolamin alleles, varietal identification and phylogenetic analysis. The method combines the benefits of HPLC, which is based on the relative affinities of different proteins/peptides to the column matrix, with MS capabilities that rely on the relative mass and velocity of various analytic components in space (vacuum).

**Figure 3:**
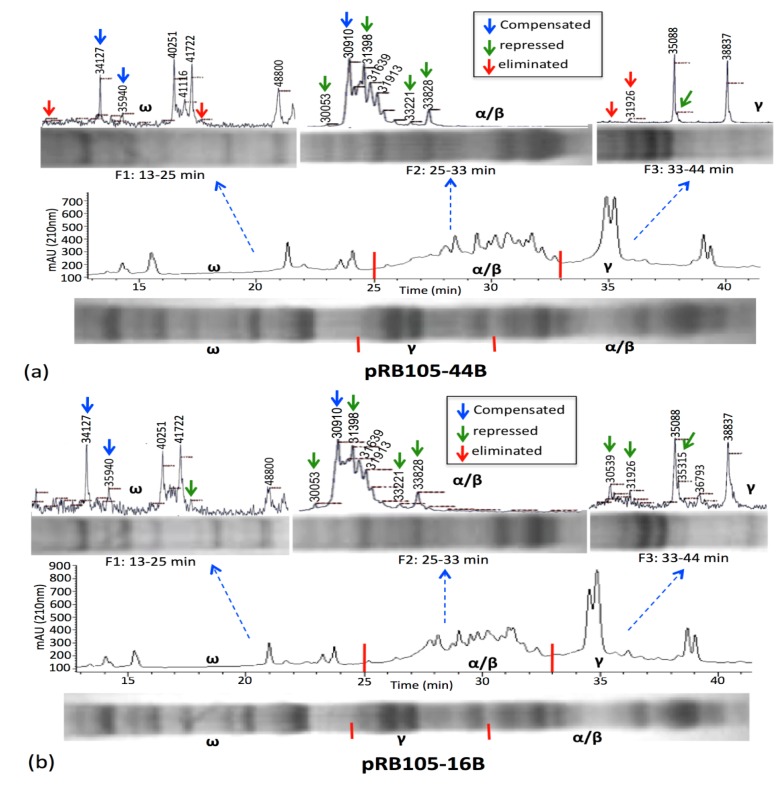
Mass spectra and electrophoresis patterns of gliadin extracts from two wheat transformants, namely pRB105-44B (**a**) and pRB105-16B (**b**). Three HPLC fractions were collected at 13–25, 25–33 and 33–44 min, and resolved on acid-PAGE as well as through MALDI-TOF-MS. Red arrows indicate complete elimination, green arrows indicate reduced accumulation, and blue arrows indicate a compensatory increase in the amount of a protein compared to the wild type control Brundage 96 (*cf.* Figure 1); F = fraction.

**Figure 4:**
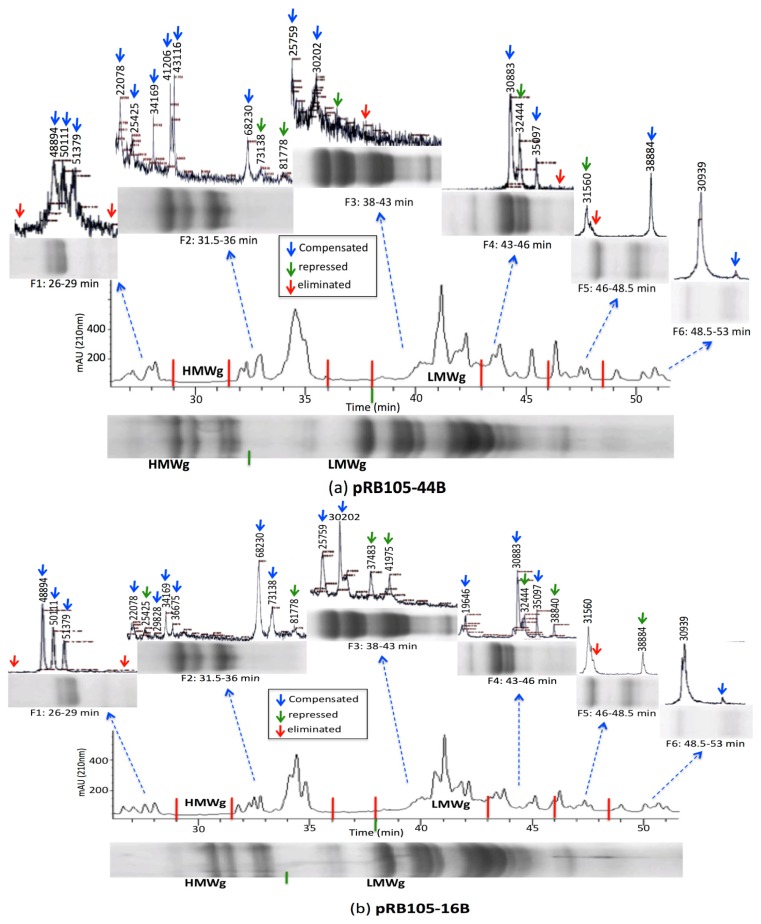
Mass spectra and electrophoresis patterns of glutenin extracts from two wheat transformants, namely pRB105-44B (**a**) and pRB105-16B (**b**). Six HPLC fractions were collected at 26–29, 31.5–36, 38–43, 43–46, 46.0–48.5 and 48.5–53.0 min, and resolved on SDS-PAGE gels as well as by MALDI-TOF-MS. Red arrows indicate complete elimination, green arrows indicate reduced accumulation, and blue arrows indicate a compensatory increase in amount of a protein compared to the wild type control Brundage 96 (*cf*. Figure 2); F = fraction.

The gluten analyses procedure developed during this study was used to analyze wheat transformants expressing artificial microRNA targeting wheat *DEMETER* homoeologues [50]. The *DEMETER* homoeologues in wheat regulate endosperm specific expression of gliadins and low-molecular weight glutenins by active demethylation of their promoters at the time of grain filling [12]. To study the epistatic effect of suppressing *DEMETER* expression on accumulation of immunogenic prolamins (*i.e*., LMW glutenins and gliadins), the T_1_ seeds of two transformants, namely pRB105-16B and pRB105-44B, respectively showing 47 and 52% suppression in *DEMETER* transcript abundance, were ground to extract gliadins and glutenins. The gliadin and glutenin extracts from the transformants were first separated respectively on acid- and SDS-polyacrylamide gels with the prolamins extracted from untransformed control Brundage 96, and later on using C8 and C18 reverse phase HPLC columns. Prolamins analyzed on the C18 RP-HPLC column using flow rates 0.2 mL/min or 1 mL/min showed limited resolution (data not shown). Similarly, the bulk gliadin and glutenin extracts analyzed using MALDI-TOF-MS resolved several differences among transformants and the wild type control, but always lacked detection of HMW glutenins, and a few other prolamins. In fact, the detection of differences was somewhat limited when the extracts were individually loaded on acid- or SDS-polyacrylamide gels and the C8 RP-HPLC column using the parameters optimized during the present study. Until the combinational gluten analysis method was adapted, the three HPLC fractions for gliadins and the six HPLC fractions for glutenins were collected and analyzed on MALDI-TOF-MS. The results of the analyses clearly showed reduced accumulation to complete elimination of a number of different prolamins in the transformants when compared with the wild type control (see Figure 3 and Figure 4). Moreover, a compensatory increase in the accumulation of a few gliadins and glutenins was also observed, which is not surprising as it was earlier reported by us using RP-HPLC in another set of wheat transformants expressing hairpin RNA against wheat *DEMETER* homoeologues [12]. The progeny of these selected transformants is currently being propagated in a greenhouse and will be reanalyzed with the procedure described above to identify the homozygous transformants. Analysis of other transformants expressing artificial microRNA is currently in process.

### 3.3. Relative Gliadin and Glutenin Concentrations Calculated Using Two Semi-Quantitative Methods

Standard curves using known quantities (2.5–300 μg/mL) of BSA were developed by densitometry and HPLC. The regression line for both standard curves was linear with *R*^2^ (correlation coefficient) values of 0.99 and 0.98 for the data obtained through densitometry and HPLC, respectively (Figure S8). Relative protein concentrations for gliadin and glutenin fractions, derived from the mature seeds of wheat cultivars Bobwhite and Brundage 96, were calculated using data from each of these platforms and by projecting them onto the respective standard curves. The estimates of relative protein concentrations using data derived from densitometry and HPLC for both cultivars and each of prolamin fractions correlated linearly with *R*^2 ^values of 0.99 in Bobwhite and 0.98 in Brundage 96 for glutenins and 0.94 in both cultivars for gliadins (Figure S9). As expected, the fractions with the lowest protein concentration calculated by densitometry also showed the lowest values using HPLC. In the case of Bobwhite, the concentration estimate for gliadins corresponded well with HPLC based estimates for each gliadin fraction, whereas the densitometric analysis of gliadin and glutenin fractions from Brundage 96 overestimated protein quantities for two gliadin fractions (13–25 min and 25–33 min) and a glutenin (26–29 min) fraction (Figure S10). Interestingly, the protein concentrations for glutenin fractions via densitometric analysis were close to twice the values obtained by the HPLC based estimates (Figure S10).

In general, protein concentrations calculated using both methods showed high correspondence with *R*^2^ values approaching 0.99. Over- or under-estimations of the protein concentrations were expected mainly due to errors introduced during manual integration of the peak areas and background noise in the gels, which could result from slight variations in staining. Moreover, the variation in the elution efficacies of BSA and specific prolamins are expected in HPLC as the elution of proteins is not perfect at any time point in the gradient of the polar and non-polar solvents, and it dependents on a number of factors including the physicochemical properties of the eluted protein(s). 

Overall, both quantification methods yielded highly related information about the relative protein quantities and thus can be interchangeably used for semi-quantitative prolamin analysis. This finding suggests that one can use labor, time and cost-effective densitometric analysis instead of HPLC for relative quantification of different prolamin family proteins without any loss of information.

## 4. Conclusions

Knowledge of protein composition in cereal grains permits breeding for genotypes based on end-use product quality and marketing parameters. Thus, improving resolution of protein analysis does not only benefit breeders in making informed selections to achieve desired end-use product quality by manipulating gluten composition through reorganization of prolamin alleles, but it will also benefit cereal chemists by providing a non-immunological method for the detection of trace amount of gluten contamination in food products. This is vital for recommending any product for consumption by celiac patients. The combinational prolamin analysis approach discussed in the present communication can potentially determine the source of contamination due to unique mass spectra of prolamins extracted from barley, wheat, rye, triticale and oats. The sequential prolamin extraction procedure that reduces the complexity of extracted fractions in combination with the two analytical platforms used in the combinational approach allow identification of prolamin contaminants in the complex food samples containing several proteins with overlapping masses. In addition, with the availability of pure prolamins from genetically engineered *E. coli* or yeast cells, it is possible to develop standard curves of these proteins using MALDI-TOF-MS and obtain quantitative and qualitative information about prolamins in a sample. This was so far not possible by MALDI-TOF-MS, due to the stochastic nature of the ionization process, which limits use of other well-characterized proteins as reference in connection with prolamin quantification.

The combinational approach is currently being used to determine the amount and type of prolamin accumulation in transformants expressing *DEMETER* silencing hairpin RNA (hpRNA) and artificial micro RNAs (amiRNAs) in the wheat endosperm. The preliminary analysis of transformants expressing hpRNA/amiRNAs demonstrated the elimination and/or reduction in the amounts of individual prolamin members or groups. This requires researchers to combine and pyramid the transformants showing complementary prolamin deficient profiles in order to achieve complete or near complete elimination of immunogenic prolamins. This is a breeding imperative since different celiac patients are sensitive to different individual prolamins. The process of selecting transformants with complementing prolamin deficiencies relies on the efficiency of the detection method. In this respect, the present communication for improvement of resolution and detection efficiency in gluten analysis by combining the benefits of the two robust detection platforms (HPLC and MALDI-TOF-MS) is essential and complements similar studies using analytical methods for characterizing prolamins [24,25,26,27,28,29,30].

## Abbreviations

RP-HPLC: reversed phase high performance liquid chromatography
LMWg: low molecular weight glutenin
HMWg: high molecular weight glutenin
MALDI-TOF-MS: matrix assisted laser desorption ionization time of flight mass spectrometry

## Supplementary Files

Supplementary File 1 Supplementary Information (PDF, 1726 KB)
