# Supplementary material for: Analysis of Wheat Prolamins, the Causative Agents of Celiac Sprue, Using Reversed Phase High Performance Liquid Chromatography (RP-HPLC) and Matrix-Assisted Laser Desorption Ionization Time of Flight Mass Spectrometry (MALDI-TOF-MS)
Source: Nutrients. 2014 Apr 15;6(4):1578–97. doi: 10.3390/nu6041578 (PMC4011052; doi:10.3390/nu6041578)
Supplement: Supplementary File 1 — Supplementary Information (PDF, 1726 KB) [file nutrients-06-01578-s001.pdf]

Supplementary Information

Figure S1. HPLC profiles of Brundage 96 and Bobwhite bulk gliadin and glutenin extracts each repeated three times to show no variation among runs. The HPLC fractions were collected from each run and combined to increase the amount of proteins in the final sample used for loading A-PAGE gels or analyzed using MALDI-TOF-MS. M = protein molecular weight marker (SM0441; Thermo Fisher Scientific Inc., Rockford, IL, USA).

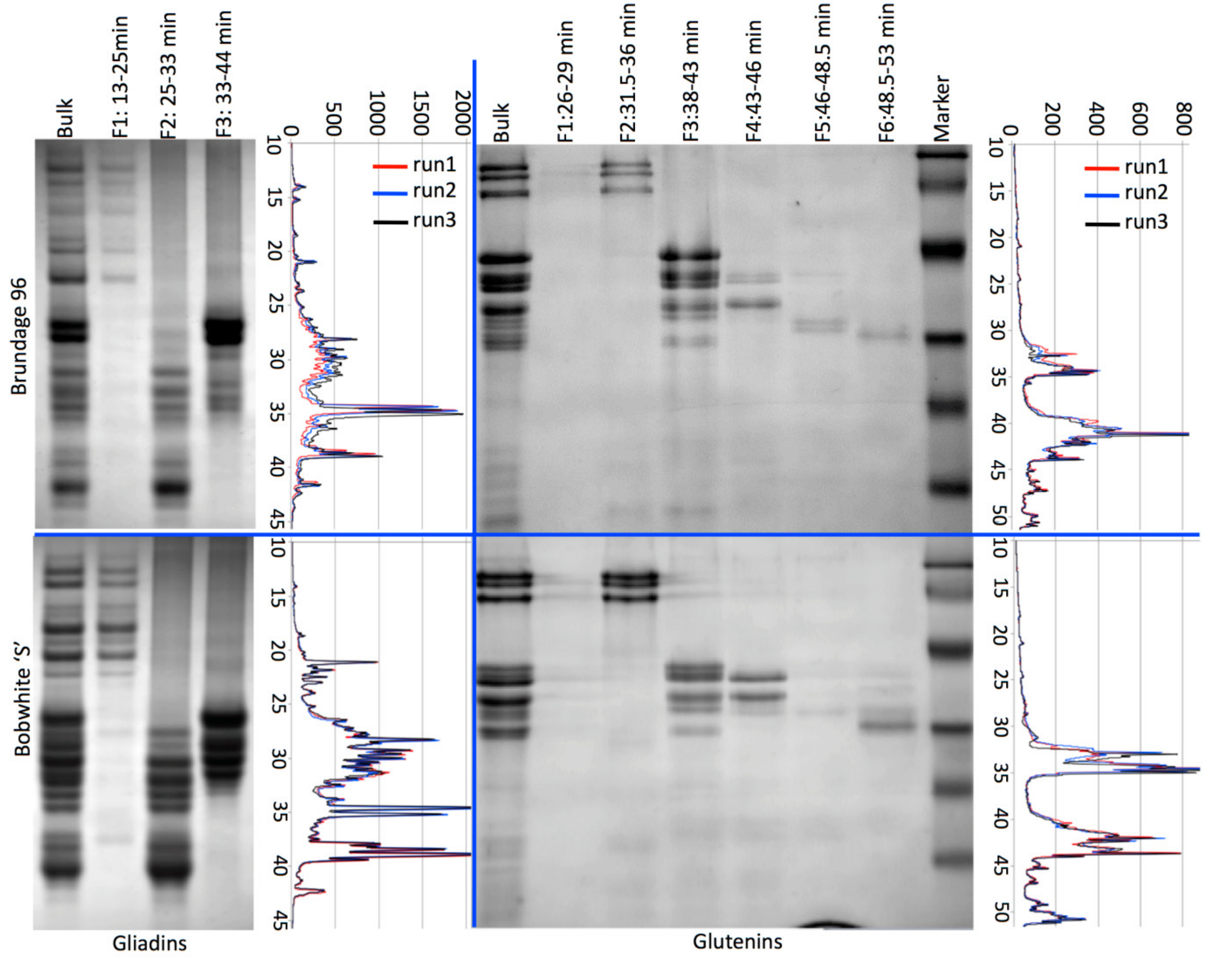

Figure S2. HPLC profiles of the bulk gliadin extract of Bobwhite obtained using Zorbax Eclipse Plus C18 and Zorbax 300SB-C8 columns. Two different flow rates were tested on the C18 column (**a**) and four different flow rates were tested on the C8 column (**b**) by injecting 30 μL of gliadins. The columns were maintained at 60 °C and applied at a gradient of 20% to 60% non-polar solvent B containing 0.1% TFA and ACN for 60 min.

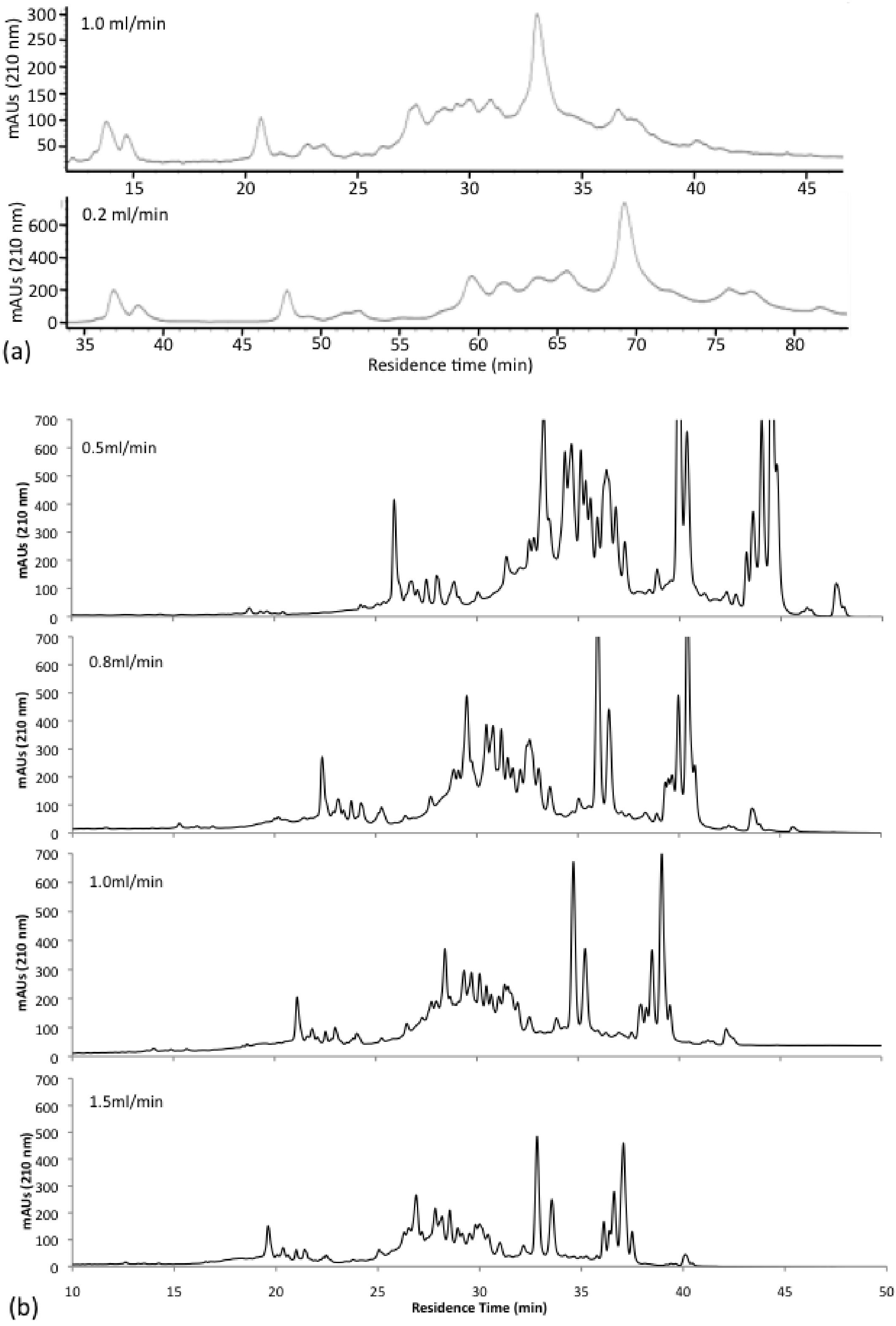

Figure S3. HPLC profiles of Bobwhite bulk gliadin extract obtained by injecting 30 µL of sample on a Zorbax 300SB-C8 column maintained at three different temperatures: 40 °C, 50 °C and 60 °C. Each run applied a gradient of 20% to 60% non-polar solvent B containing 0.1% TFA and ACN for 60 min. The peaks showing variation are marked with an asterisk and/or arrow. The *y*-axis represents absorbance in mAUs at 210 nm (not shown).

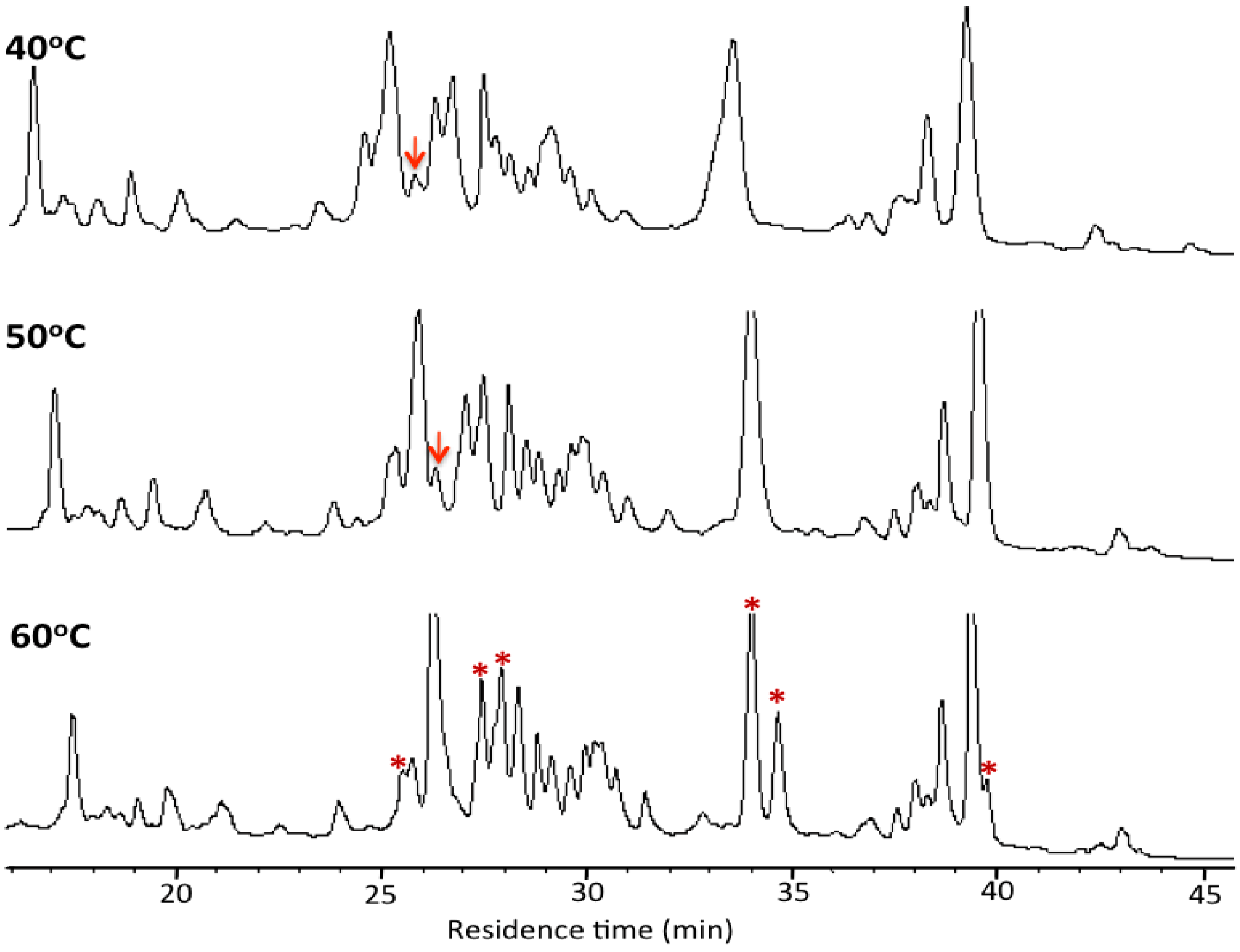

Figure S4. HPLC profiles of the Bobwhite bulk gliadin extract using two different gradients of the non-polar mobile phase solvent B containing 0.1% TFA and ACN: 10% to 80% (**a**) and 20% to 60% (**b**). In each run injected 30 µL of the sample and the Zorbax 300SB-C8 column was maintained at 60 °C. The peaks showing variation are marked with an asterisk. The *y*-axis represents absorbance in mAUs at 210 nm (units not indicated).

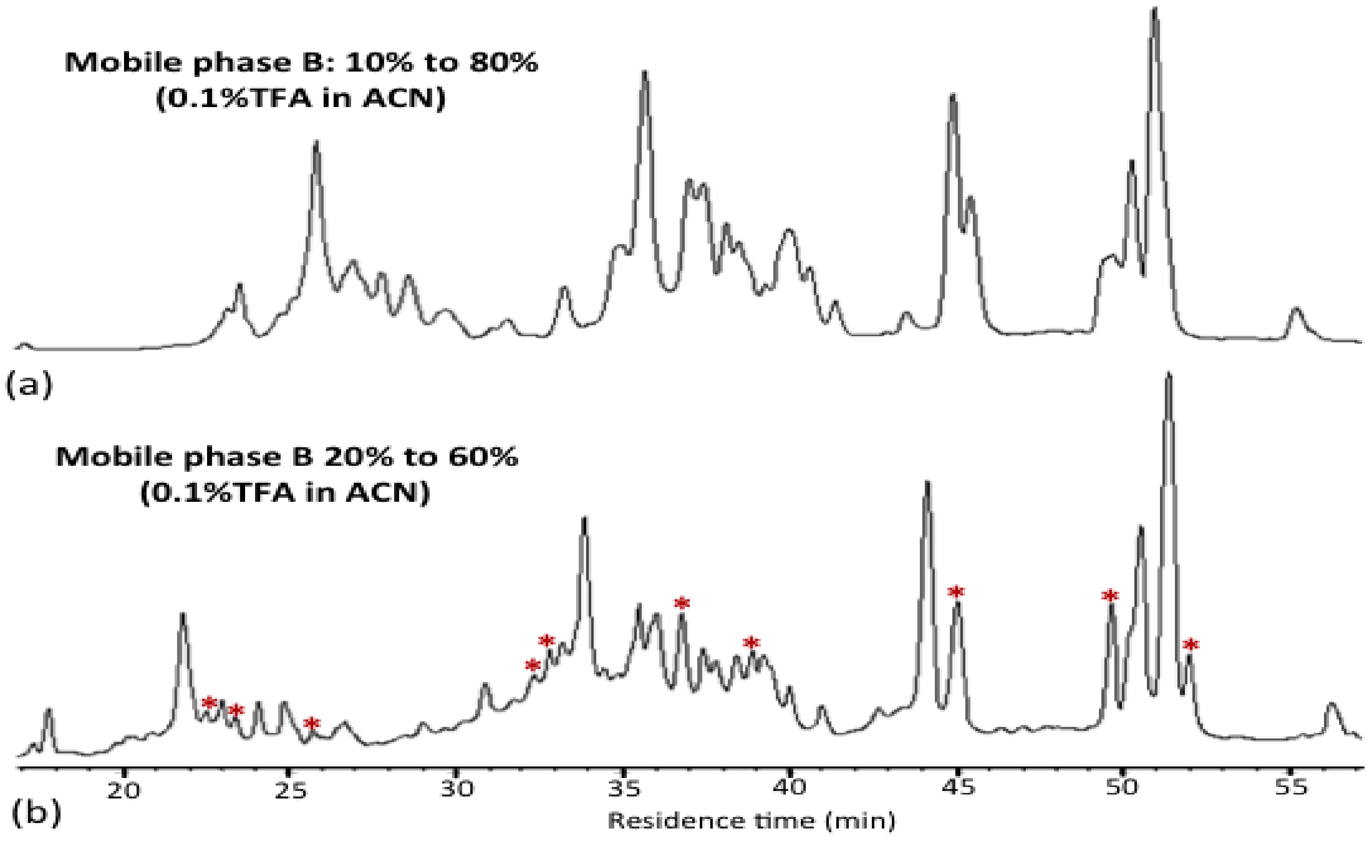

Figure S5. Mass spectrum of Brundage 96 bulk gliadin extract.

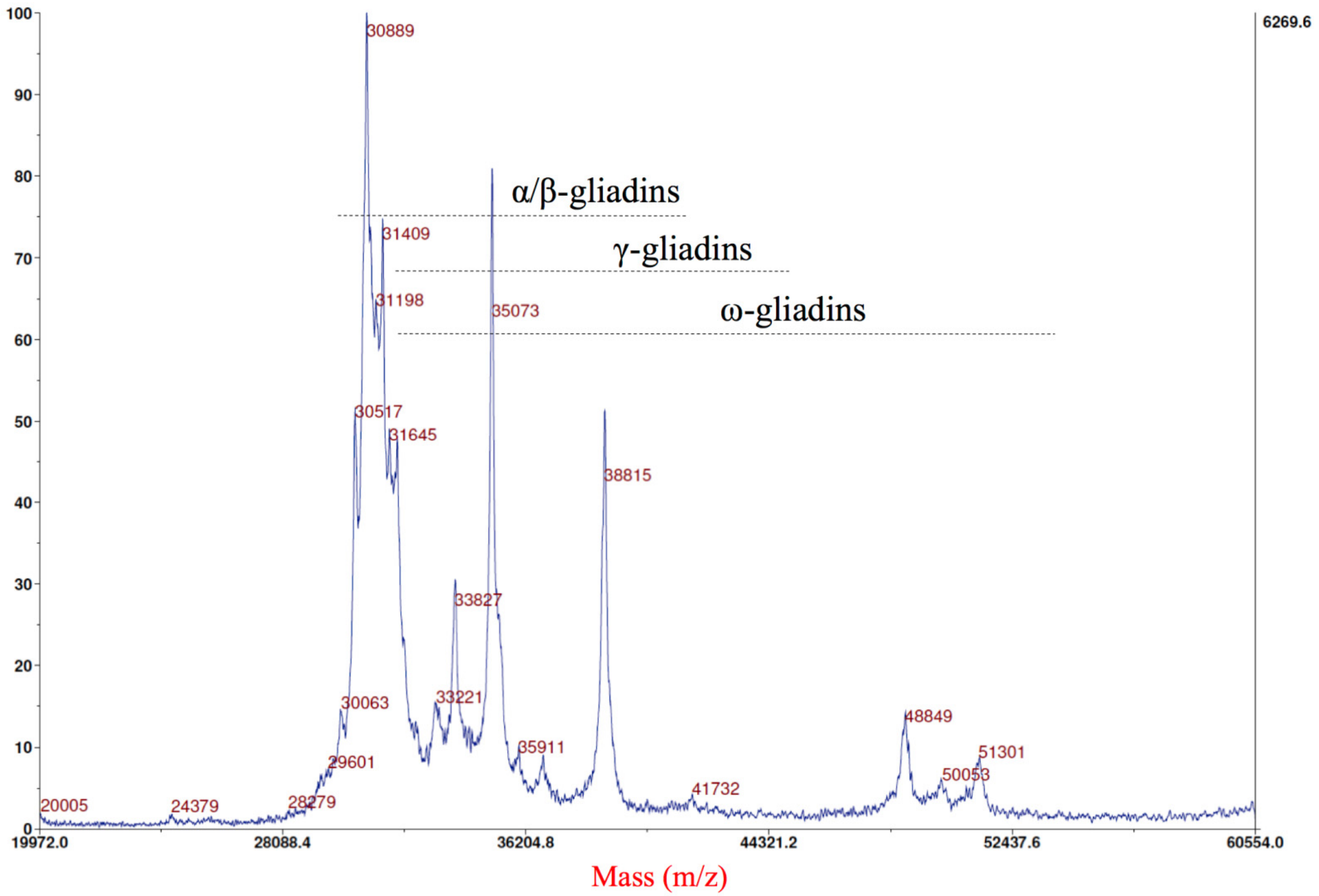

Figure S6. Mass spectrum of Brundage 96 bulk glutenin extract: in the presence of urea in the extraction buffer (**a**) and in the absence of urea in the extraction buffer (**b**).

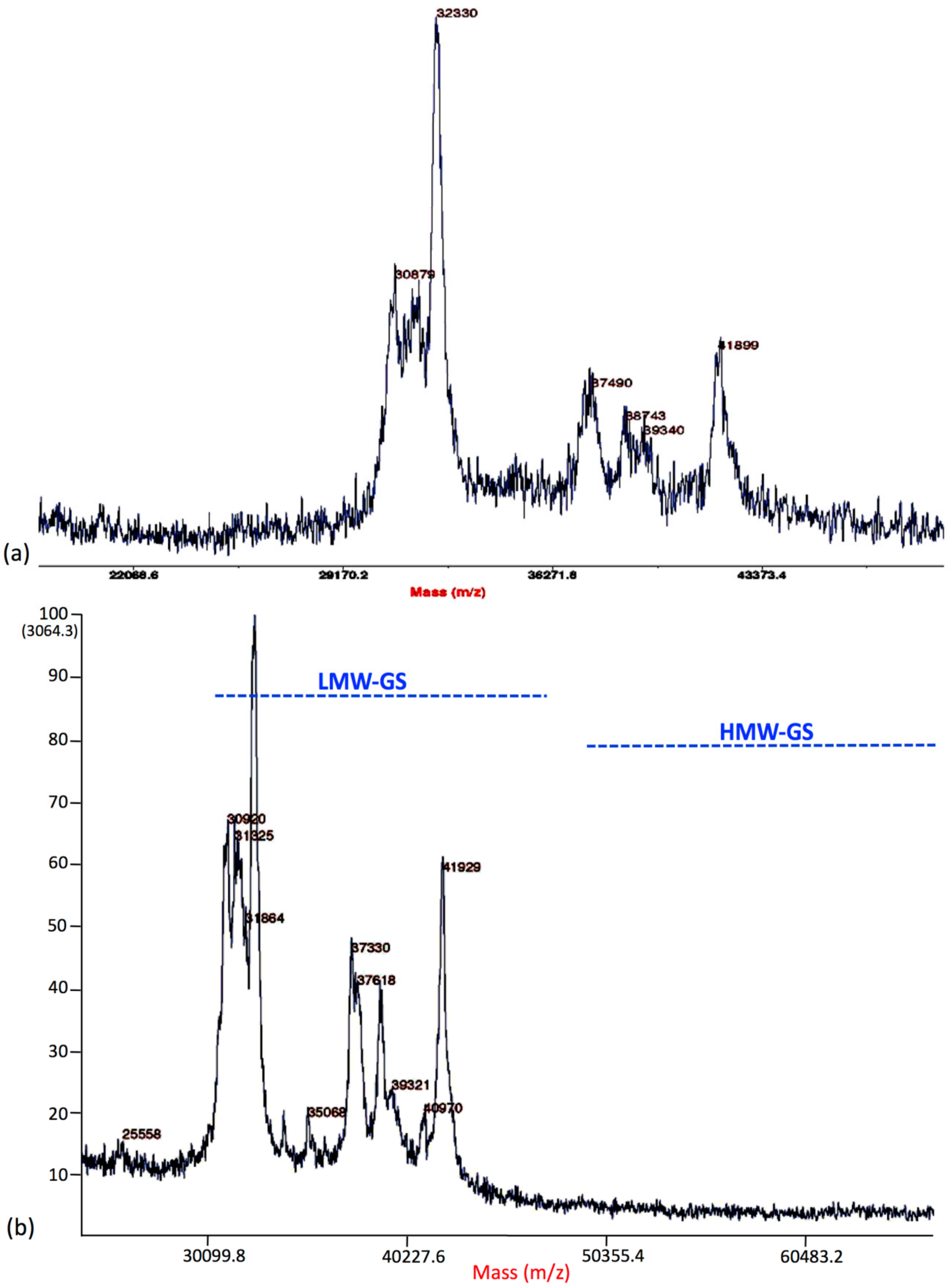

Figure S7. Glutenins extracted with buffers containing or omitting urea showed no variation in protein profiles using either SDS-PAGE or RP-HPLC. M = protein molecular weight marker (SM0441; Thermo Fisher Scientific Inc., Rockford, IL, USA).

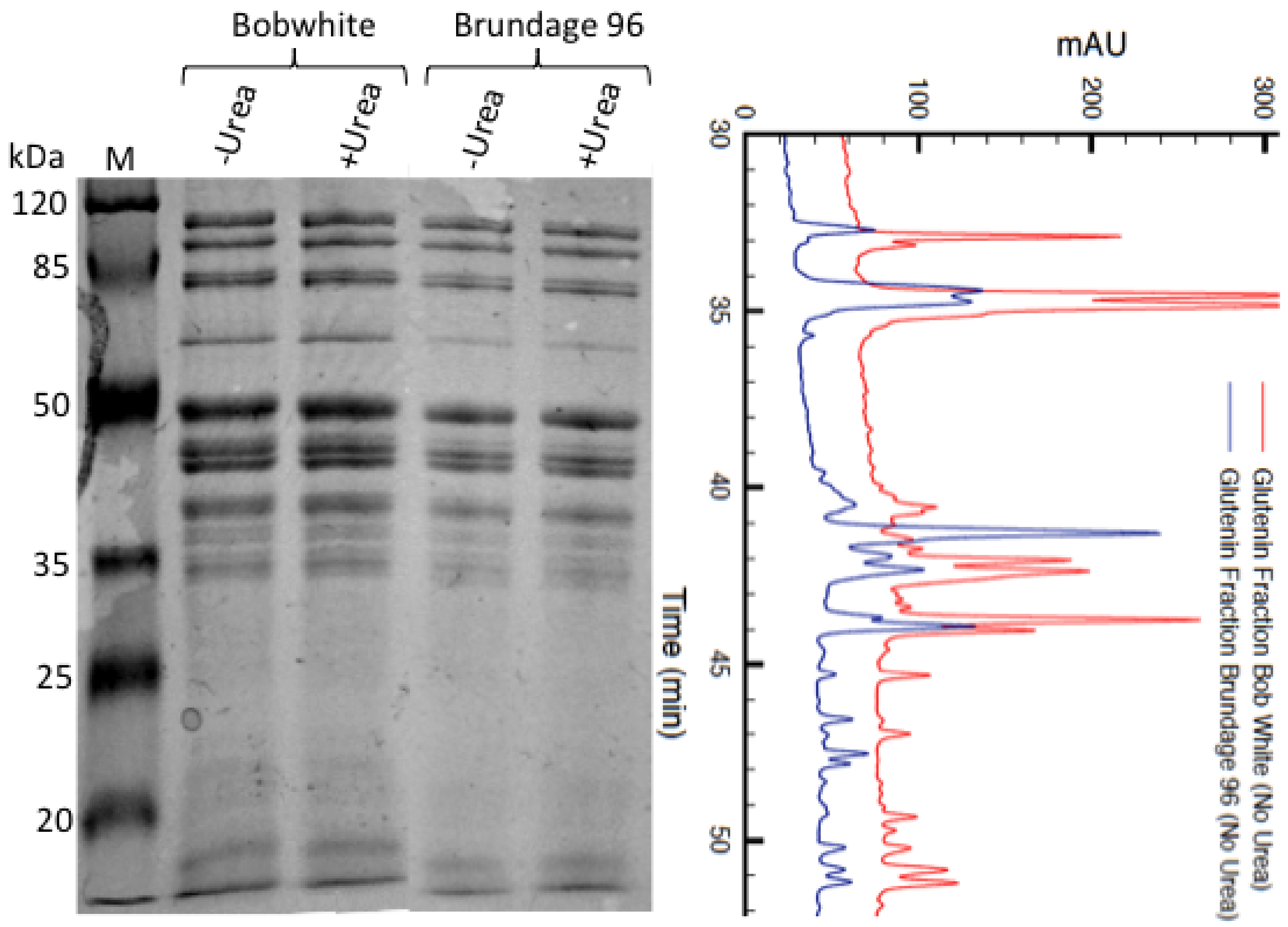

Figure S8. Standard curves prepared using known quantities (2.5 to 300 µg/mL) of bovine serum albumin using (**a**) densitometry, and (**b**) high performance liquid chromatography.

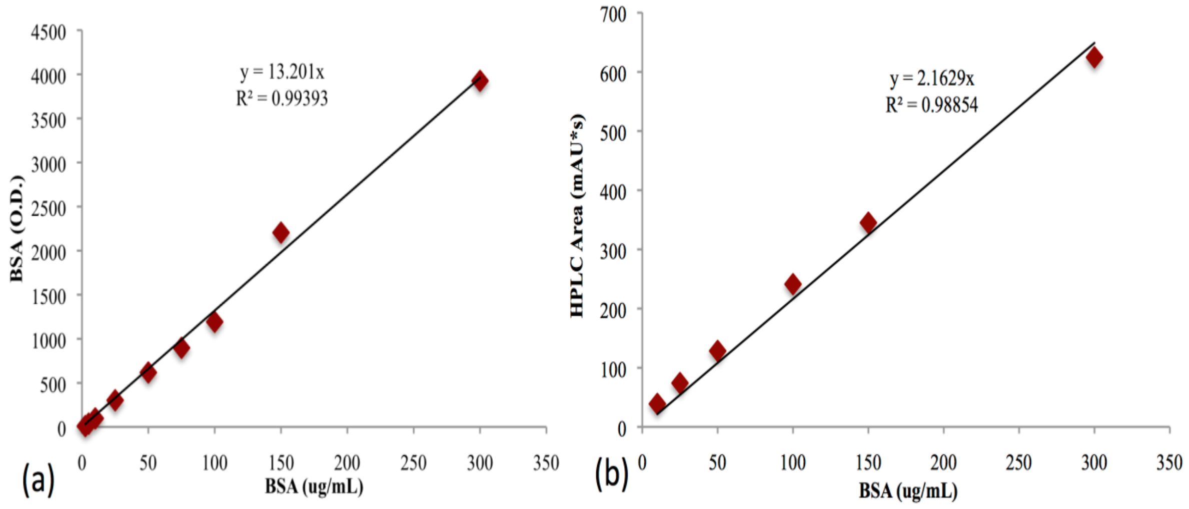

Figure S9. Regression plots showing correspondence in the relative protein quantifications made using densitometry [values recorded in optical density (O.D.)] and HPLC (values recorded in milli absorbance unit (mAU)). Glutenins and gliadins were derived from mature grains of two wheat cultivars, Bobwhite (**a**) and Brundage 96 (**b**). Each dot on the regression plot represents a prolamin fraction collected at a specific retention time from HPLC.

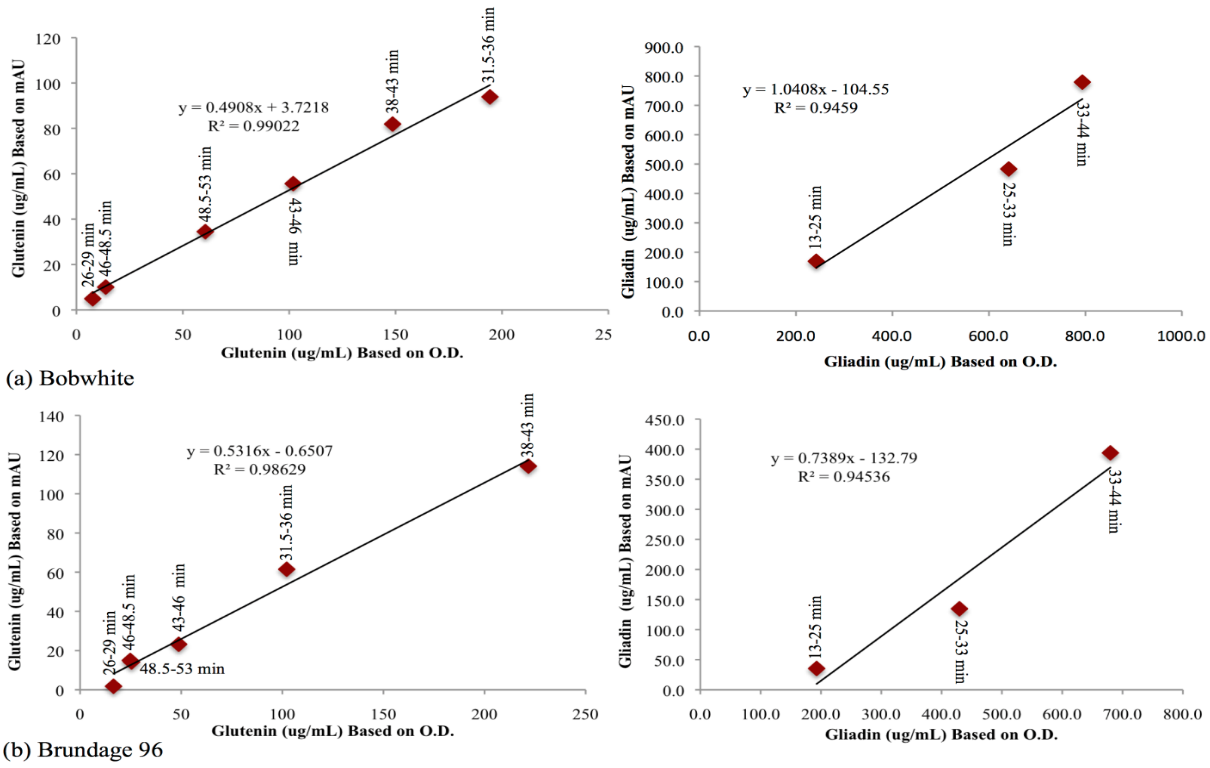

Figure S10. Comparison of relative protein quantities determined using densitometry (values recorded in optical density (O.D.)) and HPLC (values recorded in milli absorbance unit (mAU)) for each of the prolamin fraction obtained from Bobwhite (**a**) and Brundage 96 (**b**).

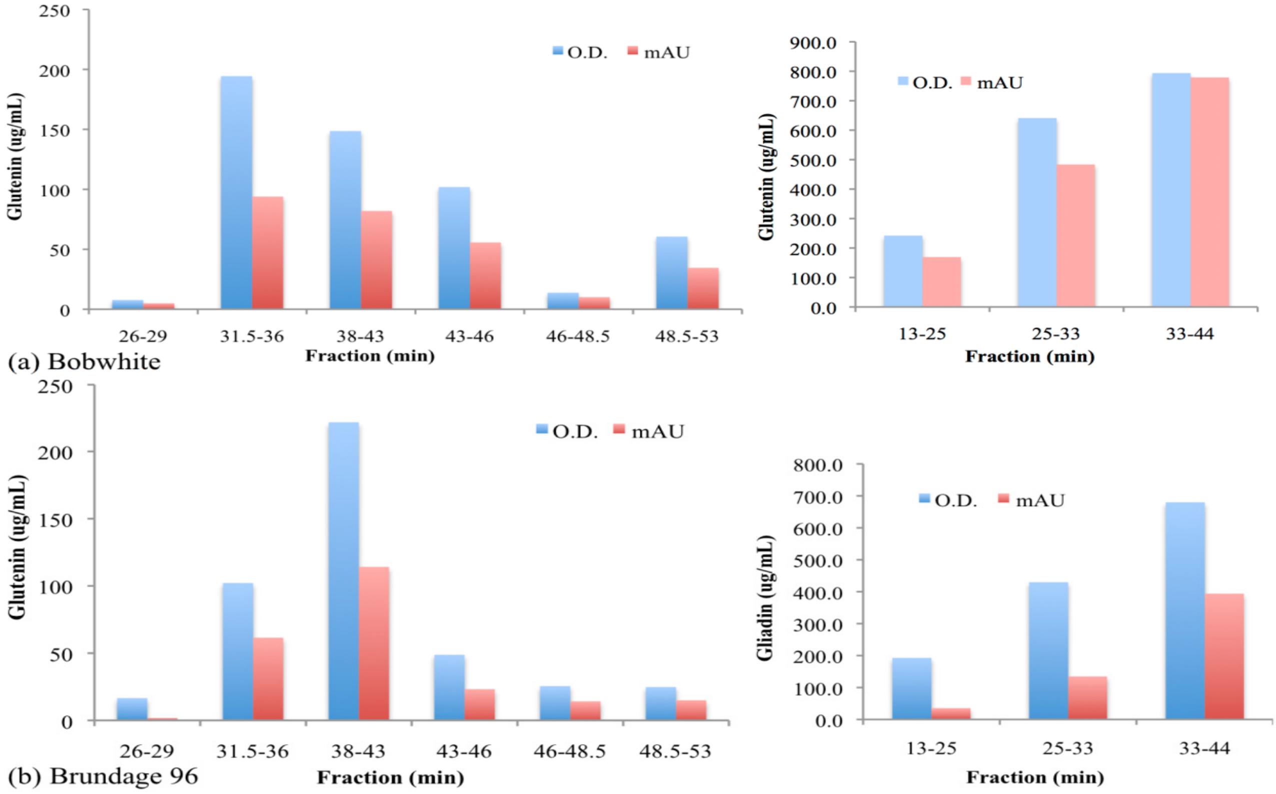

Table S1. Molecular masses of different prolamins and their proportions in the wheat endosperm.

Class	MW in kDa	Proportion (%)
	Gliadin	
α/β	28–35	28–33
γ	31–35	23–31
ω1,2	39–44	4–7
ω5	49–55	3–6
	Glutenin	
LMW-GS	32–39	19–25
x-HMW-GS	83–88	4–9
y-HMW-GS	67–74	19–25

Table S2. Mass profile of the Brundage 96 bulk gliadin extract.

Peak No.	Mass (Da)	Class
1	29,637	α/β
2	30,044	α/β
3	30,509	α/β
4	30,872	α/β
5	31,374	α/β, γ
6	31,875	α/β, γ
7	32,526	α/β, γ
8	33,221	α/β, γ
9	33,823	α/β, γ
10	35,070	α/β, γ
11	35,903	α/β, γ
12	36,731	ω1,2
13	38,811	ω1,2
14	41,744	ω1,2
15	48,837	ω5
16	51,300	ω5
17	62,752	-

Table S3. Mass profile of the Brundage 96 bulk gliadin extract.

Peak No.	Mass (Da)	Class
1	20,039	-
2	20,407	-
3	20,936	-
4	25,558	-
5	30,920	-
6	31,325	-
7	31,864	LMW-GS
8	32,306	LMW-GS
9	35,068	LMW-GS
10	37,330	LMW-GS
11	37,618	LMW-GS
12	39,320	LMW-GS
13	40,970	-
14	41,929	-

Table S4. Mass profile of Brundage 96 gliadin HPLC fractions collected at the following retention times: 13–25, 25–33 and 33–44 min.

Peak No.	Mass (Da)	Class	Peak No.	Mass (Da)	Class
	Fraction 1 (13–25 min)			Fraction 2 (25–33 min)	
1	20,070	-	1	30,061	α/β
2	20,452	-	2	30,929	α/β
3	20,592	-	3	31,394	α/β, γ
4	22,781	-	4	31,640	α/β, γ
5	25,609	-	5	31,896	α/β, γ
6	30,067	α/β	6	32,515	α/β, γ
7	30,876	α/β	7	33,202	α/β, γ
8	31,422	α/β, γ	9	33,829	α/β, γ
9	31,929	α/β, γ	10	35,095	α/β, γ
10	32,138	α/β, γ	11	38,833	ω1,2
11	33,246	α/β, γ	12	70,058	-
12	33,844	α/β, γ	13	73,748	-
13	35,158	α/β, γ	14	93,500	-
14	35,967	α/β, γ		Fraction 3 (33–44 min)	
15	40,288	ω1,2	1	29,334	α/β
16	41,127	ω1,2	2	29,639	α/β
17	41,738	ω1,2	3	29,992	α/β
18	42,617	ω1,2	4	30,525	α/β
19	48,877	ω5	5	31,838	α/β, γ
20	50,066	ω5	6	35,112	α/β, γ
21	51,337	ω5	7	35,380	α/β, γ
			8	36,781	-
			9	38,881	ω1,2

Table S5. Mass profile of Brundage 96 glutenin HPLC fractions collected at the following retention times: 26–29, 31.5–36, 38–43, 43–46, 46.0–48.5 and 48.5–53.0 min.

Peak No.	Mass (Da)	Class
Fraction 1 (26–29 min)		
1	20,033	-
2	20,345	-
3	20,963	-
4	22,582	-
5	23,446	-
6	34,305	LMW-GS
7	40,836	-
8	41,705	-
9	68,555	*y*-HMW-GS
10	87,017	*x*-HMW-GS
Fraction 2 (31.5–36.0 min)		
1	20,043	-
2	26,776	-
3	40,834	-
4	41,674	-
5	82,388	*x*-HMW-GS
6	87,005	x-HMW-GS
Fraction 3 (38–43 min)		
1	30,881	-
2	31,865	LMW-GS
3	32,293	LMW-GS
4	37,660	LMW-GS
5	41,132	-
6	42,053	-
Fraction 4 (43–46 min)		
1	30,961	-
2	32,477	LMW-GS
3	35,154	LMW-GS
4	38,866	LMW-GS
Fraction 5 (46.0–48.5 min)		
1	30,505	-
2	30,979	-
3	31,443	-
4	31,981	LMW-GS
5	32,266	LMW-GS
6	35,156	LMW-GS
Fraction 6 (48.5–53.0 min)		
1	27,403	-
2	29,426	-
3	29,951	-
4	30,939	LMW-GS
5	31,443	LMW-GS
6	31,802	LMW-GS
7	34,123	LMW-GS
8	36,850	LMW-GS

© 2014 by the authors; licensee MDPI, Basel, Switzerland. This article is an open access article distributed under the terms and conditions of the Creative Commons Attribution license (http://creativecommons.org/licenses/by/3.0/).
